# Loneliness as a determinant of healthcare utilisation in older adults: a cross-sectional study in a Portuguese rural region

**DOI:** 10.1007/s41999-025-01307-8

**Published:** 2025-10-16

**Authors:** Ângela Mira, Cristina Galvão, Paulo Santos

**Affiliations:** 1 Unidade de Saúde Familiar Alfa Beja, Unidade Local de Saúde Do Baixo Alentejo, Beja, Portugal; 2 Serviço Integrado de Cuidados Paliativos Beja+, Unidade Local de Saúde Do Baixo Alentejo, Beja, Portugal; 3 MEDCIDS—Department of Medicine of Community, Information and Health Decision Sciences, CINTESIS@RISE—Center for Clinical Investigation and Health Services Research, Faculty of Medicine, University of Porto, Porto, Portugal

**Keywords:** Geriatrics, Loneliness, Social isolation, Polypharmacy, Poverty, Family conflict, Access to primary care

## Abstract

**Background:**

Loneliness is an increasingly recognised determinant of health in older adults, associated with adverse outcomes and higher healthcare utilisation.

**Purpose:**

This study aimed to evaluate the relationship between loneliness and healthcare use in an ageing rural population in southern Portugal.

**Methods:**

A cross-sectional survey was conducted amongst community-dwelling individuals aged ≥ 65 years, randomly selected from primary care records in the Baixo Alentejo region. Data collection included the validated Portuguese version of the UCLA Loneliness Scale and self-reported use of healthcare services over the previous year (primary care and emergency visits, and the number of daily prescribed medicines).

**Results:**

A total of 318 participants were included (58.8% female), with a mean age of 75.5 years, 28.9% over 80. Mild loneliness was reported by 52.2% and severe loneliness by 14.8%. Severe loneliness was significantly associated with increased primary care visits (OR = 6.8; 95%CI: 5.8–7.7; *p* < 0.001), emergency department use (OR = 5.8; 95%CI: 4.4–7.1; *p* < 0.001), and polypharmacy (OR = 2.0; 95%CI: 1.3–2.6; *p* < 0.001). Depression, poor perceived health, family dysfunction, and urinary tract disease were associated with severe loneliness, whilst strong neighbourhood relationships appeared protective.

**Conclusion:**

Loneliness is a measurable and clinically relevant determinant of healthcare use in older adults. These findings support the integration of loneliness screening and intervention into routine clinical care, regardless of geographic setting.

## Key point messages

**Aim:**

To assess the association between loneliness and healthcare utilisation amongst older adults living in a rural region of Portugal.

**Findings:**

Mild loneliness affected over half of the participants, and severe loneliness nearly 15%. Severe loneliness was significantly associated with increased primary care visits, emergency department use, and polypharmacy, with depression, poor health perception, and family dysfunction as key predictors.

**Message:**

Loneliness is a relevant and measurable determinant of healthcare use in older adults and should be routinely addressed in clinical practice.

## Introduction

Ageing is a reality today, leading to significant social, political and health challenges, including growing loneliness [1]. It brings new problems and forces us to look for solutions based on sound evidence and the integration of healthcare providers’ experiences and patients’ values [2].

Loneliness has emerged as one of our time's most pressing and underestimated public health challenges. It is crucial to distinguish loneliness from social isolation. Whilst social isolation refers to an objective lack of social contacts or interactions, loneliness is a subjective and emotionally distressing experience resulting from a perceived mismatch between individuals’ social relationships and those they desire [3]. Thus, a person may be socially isolated without feeling lonely, and conversely, one may feel intensely lonely even when surrounded by others. In older adults, persistent loneliness is associated with increased risk of depression, cognitive decline, functional deterioration, multimorbidity, and premature mortality [1, 4, 5]. As populations around the world continue to age, loneliness has become a defining challenge of the twenty-first century, prompting experts to call it the next “geriatric giant,” on par with frailty and dementia [1].

Whilst loneliness can affect individuals across all social strata, it is most acute and consequential in rural, ageing communities where traditional family and community structures are rapidly weakening. Despite its significant burden, loneliness remains poorly recognised in clinical practice and underrepresented in health policy. Studies suggest that lonely individuals often turn to health services not due to acute medical conditions, but to seek human connection [6–8]. This reliance on healthcare to fill emotional and social voids results in increased primary care consultations, higher medication use, and more frequent emergency room visits—trends that place additional pressure on already overstretched systems and can lead to overtreatment, misdiagnosis, and preventable suffering.

In this context, our study aims to investigate the impact of loneliness on healthcare utilisation amongst older adults in a deprived and aged population. We are also interested in identifying this population’s key social, economic, and psychological factors associated with loneliness. Understanding loneliness is crucial. It highlights an urgent public health issue and contributes valuable evidence to inform national ageing strategies and international efforts to tackle social isolation amongst older adults, one of our time’s most urgent, complex, and neglected challenges [9].

## Methods

We performed a cross-sectional study by applying a questionnaire to people aged 65 or over registered in the primary healthcare settings of the *Baixo Alentejo* Local Health Unit between March 15, 2024, and August 31, 2024, representing 65,472 elders, in a total of 182,579 inhabitants. Baixo Alentejo, a rural region in southern Portugal, exemplifies this reality. It has one of the highest ageing indices in the country and a pronounced demographic imbalance with low population density, small settlements, and a significant proportion of open countryside, driven by decades of youth outmigration, economic stagnation, and geographical isolation [10, 11]. To our knowledge, this is the first comprehensive study to examine these relationships in this vulnerable region.

### Participants

The sample size was estimated at a minimum of 318 individuals, based on an expected prevalence of loneliness in the elderly of 30% [6, 12], admitting an error of 5% for a 95% confidence interval. The sample was stratified by primary healthcare centres according to the number of elders registered in each one. In each stratum, participants were randomly assigned and invited to participate. All people above 65 years old were included for randomisation. We excluded institutionalised patients, bedridden or those without the mental or cognitive capacity to respond to the survey or sign the informed consent form.

### Questionnaire

The participants were invited to fill out the questionnaire. Trained interviewers were made available to help participants respond if necessary in reading or understanding the questions asked. The questionnaire consisted of four parts: sociodemographic characterisation (age, gender, marital status, living alone, schooling, monthly income below the national minimum wage), quality of life (quality of life, participation in leisure time activities, characterisation of living with neighbours, and family functionality by familiar APGAR [13]), the perception of loneliness (16 questions, version of the Loneliness Scale from the University of California, Los Angeles (UCLA-LS)), and the characterisation of consumption of healthcare resources (number of drugs taken, number of visits to the health centre and emergency room in the last year, presence of depression by PHQ-9 questionnaire [14], comorbidities (open question), and global health status perception). Characterisation of living with neighbours and global health status were measured by a Likert scale from 0 (very low) to 10 (very high), based on individual perception. Polymedication was defined as the chronic consumption of five or more drugs a day [15].

The Loneliness Scale of the University of California, Los Angeles—UCLA -LS [16] evaluated the degree of loneliness. The Portuguese version of the UCLA-LS used in this study [17] consists of 16 items. It has two dimensions, social isolation and affinities, and presents high internal consistency in the translation and validation. An overall score of more than 32 indicates negative feelings of loneliness.

### Ethical considerations

The procedures carried out comply with the principles of the Declaration of Helsinki and the Declaration of Oviedo for the protection of human rights and the dignity of human beings in the face of the applications of biology and medicine.

The protocol for this study was approved by the Ethical Committee for Health of the *Baixo Alentejo* Local Health Unit (EDOC/2024/8768).

No identification data was collected, respecting anonymity. All participants signed the informed consent form before being included in the study. All the procedures followed the fundamental principles of medical ethics of non-maleficence, justice, autonomy and beneficence.

### Statistical analysis

The information was stored in a password-protected Microsoft™ Excel® 2021 database and analysed using the IBM SPSS® 28 software. Descriptive and dispersion measures were used to characterise the sample, calculating the frequencies of categorical and nominal variables and the mean, median and standard deviation for continuous variables according to their distribution. A multinomial logistic regression model used the backwards stepwise model to determine the weighting of each factor in mild or severe loneliness compared to those patients without loneliness. The Kruskal–Wallis test tested the association between loneliness and the impact on health services utilisation, measured by the number of medications, appointments in primary care, and access to the emergency room, due to the non-normal distribution of outcomes. The correction of Bonferroni allowed us to determine the intra-category relation. Missing data was treated as missing, without adjustment.

The association with related factors for loneliness was assessed by multinomial logistic regression between the scale categories, with the absence of loneliness as the reference. An alpha error of 5% was accepted.

## Results

We included 318 participants (58.8% female) with an average age of 75.5 years (± 7.1), 28.9% of whom were over 80.

Table 1 shows the sociodemographic characteristics of the sample. Most participants were married (64.2%), 23.0% were widowed, and 29.6% lived alone. The overall perception of their quality of life was 6.9 (± 1.7) out of 10. A monthly income of less than the national minimum wage was reported by 59.7%, and 61.3% had an education below 6 years of schooling. Most respondents had some activity in their free time (70.8%), with socialising with groups of friends and playing sports being the most frequent activities (30.5% and 25.8%, respectively). The quality of neighbourhood relations was classified at an average of 8.8 (± 1.5) out of 10, meaning good, meaningful relationships.

**Table 1:** Demographic characteristics of the sample

		Total *n* = 318	%
Sex (female)		187	58.8
Age (Mean ± SD)		75.5 ± 7.1	
Age over 80		92	28.9
Marital Status	Married	204	64.2
	Single	11	3.5
	Divorced	30	9.4
	Widowed	73	23.0
Living alone		94	29.6
Perception of quality of life (Mean ± SD)		6.9/10 (± 1.7)	
Low schooling		195	61.3
Low income		190	59.7
Leisure activities	At least one	225	70.8
	Sports	82	25.8
	Church	40	12.6
	Cultural activities	37	11,6
	Groups of friends	97	30,5
	Work activity	41	12.9
Neighbourhood coexistence scale (Mean ± SD)		8.8/10 (± 1.5)	

SD: standard deviation

In total, 166 participants presented mild loneliness (52.2%; 95%CI: 46.5–57.9%), and 47 showed severe loneliness (14.8%; 95%CI: 11.0–18.6%), according to the Loneliness Scale from the University of California, Los Angeles (UCLA). When adjusted to the 2021 Population Census, the prevalence of mild loneliness was 68.6% (95%CI: 68.1–69.1%), and severe loneliness was 19.3% (95%CI: 18.8–19.7%). There is a significant association between loneliness and the number of medications, Primary Care appointments in the last year, and visits to emergency room services (Table 2), which was maintained after adjustment for the presence of comorbidities, regardless of the disease itself.

**Table 2:** Impact of loneliness on the utilisation of health care facilities

	Without loneliness *n* (95%CI)	Mild loneliness *n* (95%CI)	Severe loneliness *n* (95%CI)	*p*-value*	*p*-value between mild and severe loneliness**	*p*-value after adjustment for comorbidities***
Number of medications	3.8 (3.4–4.3)	5.8 (5.3–6.2)	6.8 (5.8–7.7)	< 0.001	0.327	< 0.001
Number of primary care appointments	2.5 (1.8–3.2)	4.6 (3.8–5.4)	5.8 (4.4–7.1)	< 0.001	0.384	< 0.001
Number of emergency room consultations	0.5 (0.4–0.7)	0.9 (0.8–1.1)	2.0 (1.3–2.6)	< 0.001	0.007	0.010

*CI: confidence interval;* * Kruskal–Wallis test; ** Kruskal–Wallis with Bonferroni correction; *** linear regression

Table 3 shows the determinants associated with mild and severe loneliness, compared to the absence of loneliness as a reference. Ageing is a significant factor for severe loneliness but not for mild loneliness, regardless of gender. Low perception of good health status is associated with loneliness, as well as the presence of urinary tract diseases. Depression and the presence of familial dysfunction are highly related to loneliness. Conversely, low income is protective against severe loneliness. A good neighbourhood coexistence is associated with lower levels of loneliness. All other factors presented in Table 1 and included in the analysis were not significant determinants of loneliness.

**Table 3:** Significant determinants associated with mild and severe loneliness (polynomial regression compared to the absence of loneliness). Multinomial logistic regression model, adjusted for age and gender, and backwards stepwise

	Mild loneliness			Severe loneliness		
	OR	95%CI	p-value	OR	95%CI	p-value
Female	0.903	0.465–1.753	0.762	0.470	0.168–1.318	0.151
Age	0.965	0.893–1.043	0.374	1.131	1.006–1.272	0.039
Low income	0.614	0.318–1.187	0.147	0.310	0.115–0.836	0.021
Perceived health status	0.746	0.607–0.916	0.005	0.668	0.492–0.907	0.010
Urinary tract diseases	0.372	0.136–1.017	0.054	0.035	0.003–0.362	0.005
Depression	5.302	2.247–12.507	< 0.001	10.126	2.757–37.196	< 0.001
Depression affected daily activities	0.981	0.411–2.341	0.965	3.613	0.951–13.72	0.059
Familial dysfunction	7.832	0.895–68.511	0.063	22.190	2.305–213.58	0.007
Neighbourhood coexistence	0.697	0.539–0.901	0.006	0.536	0.385–0.747	< 0.001

OR: odds ratio; CI: confidence interval

## Discussion

This study reveals a deeply concerning reality: loneliness affects over two-thirds of older adults in Baixo Alentejo, with significant implications for health and healthcare utilisation, allowing us to estimate an adjusted prevalence of 88% for the total population of the *Baixo Alentejo* region, in line with another recent Portuguese study [6]. These findings are consistent with international evidence linking loneliness to higher use of health services, polypharmacy, and worse perceived health [1, 4, 6, 7]. In 2022, Surkalim et al. found an overall prevalence of loneliness in adults over 60 of 11.9% across European countries [18], with significant differences between countries despite the heterogeneity in measuring it. Stegen specifically studied community-dwelling older adults and found a prevalence of loneliness of 31.6%, with the same constraints [19]. Recently, Susanty estimated the global prevalence of loneliness in the elderly at 26%, slightly lower in Europe and Oceania, with factors like cognitive impairment, poor health, female gender, depression, widowed, single, institutionalisation, and rural residency associated with high risk [20]. The COVID-19 pandemic seems to have worsened the problem, as Su found in his review of 30 studies after the pandemic, along with increased social isolation [21].

Our study further quantifies this impact in a rural and ageing region that serves as a model for understanding similar dynamics elsewhere in Portugal and across Europe. The data clearly show that loneliness is not merely a social issue but a clinical one. Severely lonely individuals had nearly double the number of medications and significantly more appointments in both primary care and emergency departments compared to their non-lonely counterparts, not justified by the number of comorbidities or the burden of diseases. These findings support the view that, in the absence of meaningful social connection, healthcare becomes a surrogate support system. However, this tool is a costly and insufficient substitute that increases the risk of overtreatment, iatrogenesis, and resource strain [6, 8].

Our findings also reveal which specific vulnerabilities can shape loneliness. Depression, poor health perception, some comorbidities, and family dysfunction were the strongest predictors, mirroring findings in other European studies [19, 20, 22]. In contrast, strong neighbourhood relationships appeared protective, highlighting the crucial role of local, informal social ties in rural environments. This is quite different from the urban context, where this social clan hardly exists, and living alone means being really lonely [6]. In this rural environment, the relationship with neighbours allows elders to move around and fulfil themselves, suggesting that investing in social capital can be just as important as investing in healthcare infrastructure [23]. Louise C. Hawkley reached the same conclusion, pointing to the need to reduce loneliness [7], expanding this conclusion from young adulthood, meaning the need to prepare the elderly from an early age for the options each one makes throughout their lives.

Undoubtedly, health is more comprehensive than the absence of diseases or the capacity of providers to reduce their impact on people's functioning. To achieve good health and well-being of the third sustainable development goal [24], we must coordinate efforts with the educational system, social support, security and justice, environment and territorial planning, both at regional, national and international levels, including the much talked about, but little seen, health in all policies [25].

Whilst *Baixo Alentejo* is unique in its geographic and demographic characteristics [10, 11], it is emblematic of broader European trends: rural depopulation, isolation, structural ageing, and weakening social networks. As such, the region provides a valuable microcosm for informing national and international ageing strategies. Evidence from this study supports an urgent call for integrated, multisectoral policies to address loneliness as a determinant of health. Here, growing old often means growing alone. Studying loneliness in *Baixo Alentejo* is not only locally relevant. Due to its characteristics, the region acts as a sentinel territory, revealing the compounded vulnerabilities of older adults living in rural and economically disadvantaged areas. Its demographic and social profile mirrors similar regions across Portugal, southern Europe, and increasingly, post-industrial rural areas worldwide. By examining the prevalence, determinants, and health impacts of loneliness in this region, we gain insights transferable to broader populations facing similar demographic shifts.

Evidence from this study supports an urgent call for integrated, multisectoral policies to address loneliness as a determinant of health. One clear implication is the need to formally incorporate loneliness screening into routine primary care practice, particularly in rural areas. Validated tools such as the UCLA Loneliness Scale should be adopted, and healthcare professionals trained to recognise and respond to loneliness not simply as a social concern, but as a significant risk factor for clinical decline [1, 16].

In parallel, healthcare systems must move toward models that include social prescribing as structured referral pathways that connect lonely individuals with community-based activities. These may involve participation in volunteer initiatives, intergenerational programs, or neighbourhood groups designed to foster belonging and reduce isolation. International evidence suggests such approaches are cost-effective and impactful, especially when tailored to local contexts [1, 21].

Equally important is the role of local governments and municipalities in strengthening neighbourhood cohesion. Investment in public spaces, accessible transportation, and civic infrastructure can help foster informal social support networks, which are particularly crucial in rural settings like *Baixo Alentejo*, where formal services may be limited. Encouraging small-scale, community-led initiatives may be as critical as expanding institutional care [23].

Understanding the interaction between social relationships and seeking health care is crucial. Lonely patients seek the life support they need in health services. They replace the lack of meaningful relationships in their daily lives with the doctor-patient relationship, often the only person available to hear something from them and respond accordingly. However, it is difficult to schedule a visit to the doctor without a symptom to present or a sign to evaluate. It is common to present a hard complaint to justify their appointment. Unfortunately, it is also common for doctors not to have the time to dismantle the discourse and look for hidden agendas. One complaint leads to a diagnosis, and one diagnosis implies a treatment. In the end, the patient suffering from the social diagnosis leaves the consultation with a new illness, which is something palpable and embodied, justifying several other appointments despite the potential for iatrogenesis and for inducing actual disease in a real, never-ending cycle. Even when doctors suspect it, they rarely counter it because of the lack of articulation between health services and other relevant agents, making it difficult to be consistent with the true diagnosis, as it lacks the proper treatment. In this sense, health services must collaborate closely with social care, education, housing, and local governance under the principles of the World Health Organisation’s "Health in All Policies" framework. A coordinated approach is essential to create a cross-sectoral collaboration to address the systemic roots of loneliness and to reverse the unintended medicalisation of social suffering [25].

Finally, there is a strong case for prevention across the life course. Social resilience should begin early, through educational programs that foster community engagement, interpersonal skills, and preparation for ageing. Promoting lifelong learning, civic participation, and intergenerational contact can reduce the cumulative risk of loneliness in later life, aligning with growing calls for a more socially inclusive vision of healthy ageing [7, 8]. We excluded the institutionalised people, where the situation is still more disturbing because of the association with specific diseases such as depression, suicidal ideation, and frailty [22].

This study has limitations that warrant consideration. The cross-sectional design prevents causal inferences, and selection bias may have occurred since only individuals attending primary care were included. Recall and social desirability bias may have influenced self-reported variables such as service utilisation and family dynamics. Furthermore, the binary income variable may inadequately capture perceived economic vulnerability. Nevertheless, these constraints are not relevant enough to alter our conclusions. Still, the use of validated instruments (e.g. UCLA-LS, PHQ-9, APGAR) and a stratified random sample across primary care centers enhances internal validity and ensures broad representation of the older rural population.

Addressing loneliness demands more than compassion. It requires a system change. In rural, ageing regions like Baixo Alentejo, this change must come through decentralised, community-based solutions that prioritise human connection as a core component of health. Only then can we reduce avoidable healthcare utilisation, promote healthy ageing, and meet an increasingly invisible population’s social and emotional needs.

## Conclusions

This study reinforces the critical relationship between loneliness and healthcare utilisation amongst elders, highlighting the complex interplay between social determinants and clinical outcomes. Loneliness emerges as a significant determinant of health, often leading to increased reliance on medical services as a substitute for meaningful social relationships.

Addressing loneliness requires a paradigm shift in healthcare delivery. Medical professionals must be equipped to identify social diagnoses without overmedicalizing symptoms, work with other players to manage these patients effectively, and find the best treatment they need holistically. Establishing protocols that bridge health services and social support systems is mandatory to provide comprehensive care for lonely elders and to understand that problems do not arise in old age. Individuals must be prepared from early life stages for social resilience in later years through education and community engagement in true healthy ageing throughout life.

Loneliness is then a determinant of health, impacting people and making them suffer and feel sick. When present, it must be managed like any other risk factor. However, it can be prevented. We will certainly have to focus our attention now and in the future, both at the individual and systemic levels, to improve elder well-being, reduce healthcare costs, and align with broader public health goals.

## Data Availability

The database is under the custody of the authors in a protected archive. It may be made available directly to the authors upon request.
